# The role of organizational research in implementing evidence-based practice: QUERI Series

**DOI:** 10.1186/1748-5908-3-29

**Published:** 2008-05-29

**Authors:** Elizabeth M Yano

**Affiliations:** 1 Veterans Affairs (VA) Health Services Research and Development (HSR&D) Center of Excellence for the Study of Healthcare Provider Behaviour, VA Greater Los Angeles Healthcare System, Sepulveda, CA, USA; 2 Department of Health Services, UCLA School of Public Health, Los Angeles, CA, USA

## Abstract

**Background:**

Health care organizations exert significant influence on the manner in which clinicians practice and the processes and outcomes of care that patients experience. A greater understanding of the organizational milieu into which innovations will be introduced, as well as the organizational factors that are likely to foster or hinder the adoption and use of new technologies, care arrangements and quality improvement (QI) strategies are central to the effective implementation of research into practice. Unfortunately, much implementation research seems to not recognize or adequately address the influence and importance of organizations. Using examples from the U.S. Department of Veterans Affairs (VA) Quality Enhancement Research Initiative (QUERI), we describe the role of organizational research in advancing the implementation of evidence-based practice into routine care settings.

**Methods:**

Using the six-step QUERI process as a foundation, we present an organizational research framework designed to improve and accelerate the implementation of evidence-based practice into routine care. Specific QUERI-related organizational research applications are reviewed, with discussion of the measures and methods used to apply them. We describe these applications in the context of a continuum of organizational research activities to be conducted before, during and after implementation.

**Results:**

Since QUERI's inception, various approaches to organizational research have been employed to foster progress through QUERI's six-step process. We report on how explicit integration of the evaluation of organizational factors into QUERI planning has informed the design of more effective care delivery system interventions and enabled their improved "fit" to individual VA facilities or practices. We examine the value and challenges in conducting organizational research, and briefly describe the contributions of organizational theory and environmental context to the research framework.

**Conclusion:**

Understanding the organizational context of delivering evidence-based practice is a critical adjunct to efforts to systematically improve quality. Given the size and diversity of VA practices, coupled with unique organizational data sources, QUERI is well-positioned to make valuable contributions to the field of implementation science. More explicit accommodation of organizational inquiry into implementation research agendas has helped QUERI researchers to better frame and extend their work as they move toward regional and national spread activities.

## Background

Health care organizations exert significant influence on the quality of care through an array of factors that directly or indirectly serve as the context in which clinicians practice and patients experience care [1]. A greater understanding of this context can be important in closing the gap between research and practice. Each health care setting into which innovations are introduced represents its own organizational milieu, such as the structure and processes that comprise how an organization operates and behaves. Individually or in combination, these structures (e.g., size, staffing) and processes (e.g., practice arrangements, decision support) have the potential to foster or hinder discrete steps in the adoption and use of new technologies, care arrangements, and quality improvement (QI) strategies. Fixsen and colleagues describe such variables as being "like gravity...omnipresent and influential at all levels of implementation" [2]. Unfortunately, much implementation research has failed to fully recognize or adequately address the influence and importance of health care organizational factors, which may compromise effective implementation of research into practice [3].

Evaluating the organizational context for delivering evidence-based practice is a critical adjunct to efforts to systematically improve quality. This paper uses the context of and examples from the U.S. Department of Veterans Affairs (VA) Quality Enhancement Research Initiative (QUERI) to illustrate a framework for fostering the integration and evaluation of health care organizational factors into the planning and study of the implementation of evidence-based practice within the context of the six-step QUERI model. Based on implementation experiences since QUERI's inception, we describe the role of organizational research using a series of QUERI-specific applications. We also briefly examine the contributions of organizational theory and environmental context to the organizational research framework.

This article is one in a *Series *of articles documenting implementation science frameworks and approaches developed by the U.S. Department of Veterans Affairs (VA) Quality Enhancement Research Initiative (QUERI). QUERI is briefly outlined in Table 1 and is described in more detail in previous publications [4,5]. The *Series' *introductory article [6] highlights aspects of QUERI related specifically to implementation science and describes additional types of articles contained in the *QUERI Series*.

**Table 1:** The VA Quality Enhancement Research Initiative (QUERI)

The U.S. Department of Veterans Affairs' (VA) Quality Enhancement Research Initiative (QUERI) was launched in 1998. QUERI was designed to harness VA's health services research expertise and resources in an ongoing system-wide effort to improve the performance of the VA healthcare system and, thus, quality of care for veterans.

QUERI researchers collaborate with VA policy and practice leaders, clinicians, and operations staff to implement appropriate evidence-based practices into routine clinical care. They work within distinct disease- or condition-specific QUERI Centers and utilize a standard six-step process:

1) Identify high-risk/high-volume diseases or problems.
2) Identify best practices.
3) Define existing practice patterns and outcomes across the VA and current variation from best practices.
4) Identify and implement interventions to promote best practices.
5) Document that best practices improve outcomes.
6) Document that outcomes are associated with improved health-related quality of life.

Within Step 4, QUERI implementation efforts generally follow a sequence of four phases to enable the refinement and spread of effective and sustainable implementation programs across multiple VA medical centers and clinics. The phases include:

1) Single site pilot,
2) Small scale, multi-site implementation trial,
3) Large scale, multi-region implementation trial, and
4) System-wide rollout.

## Methods

Using the six-step QUERI process as a foundation (Table 1), we designed an organizational research framework to help improve and accelerate implementation of evidence-based practice into routine care. We reviewed organizational research from specific QUERI Centers, culling and summarizing the organizational measures they included and the methods used to apply them to different implementation research efforts. We describe these applications in the context of a continuum of organizational research activities to be conducted before, during and after implementation.

## Role of organizational factors in the QUERI model of implementation research

Evaluation of the influence of organizational characteristics on the quality of care has gained in its salience and value, as efforts to implement evidence-based practice into routine care have grown [7], although with mixed results [8]. As interventions to improve quality through structured implementation programs have moved from relatively homogenized "ideal" clinical settings to more diverse clinical environments, where tight research controls may be replaced by handoffs to hospital and practice teams, the organizational context becomes increasingly central to our understanding of what works and does not work in implementing research-defined structures and processes into operational realities [9,10]. Historically, since most clinical and delivery system interventions have been tested in a single or small number of institutions, within which the efficacy of the intervention has been evaluated and honed, organizational conditions have been either ignored (since they assumedly did not vary) or somehow controlled for. As a result, relatively few linkages between organizational structure and quality (either processes or outcomes of care) have been demonstrated [11]. However, as these clinical and delivery system interventions are implemented in more organizations in diverse settings and in different locales, the ability to implement them in the manner in which they were originally defined and demonstrated to be effective will continue to decline without better and more explicit integration of an organizational research framework into implementation research agendas [12]. As the need to adapt implementation efforts to local circumstances is increasingly recognized, the value of collecting advance information about structural and process characteristics in target institutions also has become more prominent [13].

The mechanisms by which organizational structures and processes may influence quality operate at many levels, and as a result, conceptualizations of what is meant by the organization of a health care system, setting or practice vary [14]. The diversity of how health care organizational factors are framed and defined complicates their measurement and the ability to easily integrate them into efforts to improve quality of care. How individual organizational constructs are conceptualized and measured in relation to implementation research efforts depends, in large part, on the following:

• The conceptual model and organizational theory (or theories) underlying the research [15];

• The nature of what is known and/or being hypothesized about the organizational structures and processes underlying evidence-based care for each condition under study [16];

• The size and complexity of the organization itself, such that it is clear whether we are talking about a team, a practice, a network of practices, a system of multiple networks, or some other organizational configuration;

• The timing or stage of implementation during which organizational research is being conducted (i.e., as part of planning, during implementation to support adaptation and midcourse corrections, or after implementation in support of interpretation of findings, sustainability and spread) [13]; and,

• The nature of the study designs and evaluation methods needed to demonstrate implementation effectiveness and foster sustainability and spread at the organizational level.

### Organizational theory and conceptual frameworks

To date, the use of organizational theory in the design and deployment of evidence-based practices into routine care has been highly variable and generally under-used [17]. The dilemma for many implementation researchers is the absence of clear guidance on the nature of key theories and how best to use them [18]. QUERI is no different in this regard. Thus far, QUERI researchers have chiefly adopted useful heuristic models and conceptual frameworks (e.g., Greenhalgh's model, PRECEDE-PROCEED, RE-AIM, Chronic Care Model, complex adaptive systems), organizing measures around general constructs – but not necessarily grounding them in organizational theory [19-23]. New paradigms are needed that integrate salient psychological and organizational theories into a uniform model and make them accessible to implementation researchers [24,25]. In the absence of such paradigms, implementation researchers should capitalize on the contribution of organizational theories already contributed by psychology, sociology, management science and other disciplines in order to be explicit about the anticipated mechanisms of action at the organizational level. For example, these include diffusion theory, social cognitive and influence theories, the theory of planned behaviour, as well as institutional, resource dependency, and contingency theories [24,26-28].

### What is known about organizational structures and processes underlying evidence-based practice

The Cochrane Effective Practice and Organization of Care (EPOC) group has conducted systematic reviews of a broad array of organizational and related professional practice interventions [29]. While there is a relative plethora of strategies, programs, tools and interventions in the literature about ways to improve quality, the evidence base for systematically transforming care using established interventions is actually relatively poor [30], particularly in relation to the "black box" of organizational attributes. Outside of QUERI, organizational strategies for hospital-based quality improvement (QI) have included data systems for monitoring, audit-and-feedback, and decision-support functions; financial support for QI; clinical integration; information system capability such as electronic medical records; [31], as well as compensation incentives [32]. Organizational culture as an intervening attribute has had mixed results, with greater influence on the effect of organizational strategies [33], and limited if any influence in physician organizations [34]. Practice individuation or tailoring also has had variable success [35-37].

### Timing of organizational research applications before, during and after implementation

When to introduce organizational research applications as an adjunct to implementation efforts also has not been well-described. First, organizational factors may be broadly applied as a pre-step to the design of QI interventions by elucidating organizational precursors of high and low performance [37], or more narrowly applied in preparation for refining an implementation strategy in one or more specific facilities via needs assessment [13]. During implementation, attention to local organizational structures and processes enables systematic assessment of their influences on fidelity to the evidence (e.g., is the care model being deployed in ways consistent with the evidence base?). Such assessments may be accomplished through qualitative and quantitative methods. Such organizational assessments are sometimes used as an integral function of evaluating implementation in real time to enable mid-course corrections through audits, feedback, and adjustment of intervention elements (formative evaluation) [38], and other times as post-implementation appraisals.

If done iteratively, as in the Plan-Do-Study-Act (PDSA) cycles of individual quality improvement (QI) projects, local adaptation and resolution of implementation problems at the organizational level may be accelerated. Traditionally applied in continuous quality improvement (CQI), PDSA cycles are generally designed to take a single or few patients or providers through a series of processes underlying a proposed QI activity to iteratively test what works or does not work before investing in widespread policy or practice change [39]. Each process is refined, and new elements are added or others subtracted until the complete set of actions is found to be effective in a particular setting. In implementation research, PDSA cycles offer the same opportunity to hone implementation strategies in diverse settings. The system level PDSA occurs when the PDSA cycles move from implementation within a single organization to a set of organizations that may or may not be similar in characteristics to the original institution [13]. Such system-level PDSA cycles are consistent with Phase 2 (i.e., modest multi-site evaluations) or Phase 3 (i.e., large-scale adoption programs) implementation projects in the QUERI pipeline [6]. Not all QUERI Centers have relied on PDSA approaches for their implementation efforts. However, as more of them move to multi-site implementation trials or are engaged in regional or national spread initiatives, we anticipate that greater appreciation of the details needed to adapt evidence-based practices to different organizational contexts will be helpful.

After implementation ends, traditional process and outcomes evaluations may be augmented with analyses of organizational variations in implementation strategies and outcomes (e.g., system-level effectiveness or costs) and the degree to which organizational factors influence sustainability and spread. Examining the impacts of the newly implemented evidence-based care on the organization as a whole is also an essential evaluation component as they begin to form the foundation for a business case for quality improvement for health care managers. Such a business case might include changes in performance measures, employee satisfaction/retention, or evidence for the organizational return-on-investment associated with changes in care [40,41]. Systematic collection, analysis and reporting of detailed organizational data may then contribute to updated guidelines that integrate effective adaptations for different organizational characteristics.

### Study designs and evaluation methods supporting implementation effectiveness

Achieving study designs and methods that produce credible evidence with relevance to "real world" settings is challenging, especially when aiming to evaluate population-based or practice-level interventions [42,43]. Balancing the needs of internal and external validity, pragmatic clinical trials offer participating sites an opportunity to modify the intervention to a degree that is likely to mirror what would happen under routine-care implementation [44,45]. Rather than open the "black box," these trials assume that the known (and unknown) variables are randomly distributed between intervention and control sites. Systematically assessing organizational factors through qualitative or quantitative methods may nonetheless provide a useful empirical complement to our use of pragmatic clinical trials. This is especially true in circumstances when researchers have reason to believe the variables of interest are not, in fact, randomly distributed. These types of data also are likely to improve our understanding of factors that influence provider or site participation [46,47] and the nature of modifications that worked in different organizational contexts [48].

Ensuring integration of rigorously designed and well-conducted organizational research to the mix will require not only broader recognition of its contribution to the goals of implementation science, but also an organizational research framework, like the one proposed here, that guides researchers to the types of organizational research they ought to be considering each step along the way. We posit that collecting and using organizational data will increase what we are able to learn about what settings, arrangements and resources foster or hinder adoption, penetration, sustainability and spread beyond the trial or implementation process. As Green and Glasgow suggest, "If we want more evidence-based practice, we need more practice-based evidence" [49].

## Common concepts representing health care organizational factors

Several common concepts have been used to describe the characteristics of health care organizations (Table 2). For the purposes of generally classifying different types of organizational attributes related to quality of care, we delineate them along the lines of Donabedian's structure, process and outcome framework [50].

**Table 2:** Common measures of the characteristics of health care organizations

**Organizational structure**
• Size of organizational unit(s) (e.g., facilities, beds, providers)
• Academic affiliation (e.g., scope of training programs, integration of trainees in care delivery)
• Service availability (e.g., differentiation and scope of services, general and specialty services, access to specialized units)
• Configuration (e.g., service lines, teams, integrated networks)
• Staffing/skill-mix (e.g., types of providers, level of training/experience)
• Leadership structure/authority (e.g., leadership quality, hierarchical vs. vertical structures, ownership, practice autonomy, organizational influence)
• Financial structure (e.g., health plan, reimbursement, compensation structures)
• Availability of basic and specialized service, equipment or supplies
• Resource allocation methods, resource sufficiency, and equitable distribution
• Organizational culture (e.g., group culture, teamwork, risk-taking, innovativeness)
• Work environment/organizational climate
• Knowledge, attitudes, beliefs of managers, providers, staff (e.g., organizational readiness to change)
• Level of organizational stress/tensions, degree of hassles

**Organizational Processes**

• Care management processes (e.g., practice arrangements, use of care managers to coordinate services and follow-up)
• Referral procedures (e.g., demonstration of need for referral, identification of appropriate provider resources, nature of handoffs, communication of referral results/outcomes, returns)
• Organizational supports for clinical decision-making (e.g., use of reminders, disease-specific checklists or computerized templates, electronic co-signing; designated staff implementing general or disease-specific protocols)
• Recognition/rewards, incentive systems, pay-for-performance
• Communication processes, procedures, quality of interactions
• Relationships (nature of roles and responsibilities, interpersonal styles,)
• Problem solving, conflict management, communication and response to expectations

**Organizational Outcomes**

• Process quality measures (e.g., percentage of eligible diabetics receiving foot sensation exams)
• Intermediate outcome measures (e.g., glycemic control among diabetics in the entire practice)
• Disease-related outcomes (e.g., complication rates, disease-specific morbidity and mortality)
• Global health status measures (e.g., functional status)
• Utilization measures (e.g., ambulatory care sensitive admission rates, guideline-recommended use of services at the organizational level)
• Workflow or efficiency measures (e.g., wait times, workload)
• Costs (e.g., costs of the QI intervention and its implementation at the organizational level)

*Organizational structures *tend to focus on static resources, whether they are related to the physical plant (e.g., amount of clinical space); the functions of care incorporated into the physical plant (e.g., types of specialized units); the equipment they contain (e.g., availability of laboratory or diagnostic equipment, machinery, computers); or the people employed to deliver services (e.g., staffing levels, skill mix) [50]. These facets may be described as the health care infrastructure, and while they can be changed, they are not typically as mutable as other characteristics [51,52]. Governance, managerial or professional arrangements for overseeing, managing and delivering services (e.g., corporate leadership structures, types of health plan, service lines, and health care teams) also represent structural measures [53-55]. The diffusion of innovation literature portrays these measures as "inner context," pointing to greater assimilation of innovations in organizations that are large (likely a proxy for slack resources and functional differentiation), mature, functionally differentiated (i.e., divided into semi-autonomous departments or units), and specialized (i.e., sufficient complexity representing needed professional knowledge and skill-mix) [19].

*Organizational processes *may be distinguished from the classical interpretation of Donabedian's process of care measures by virtue of their role in supporting the actions between provider and patient at a given encounter [50]. While they are influenced by organizational structure, they tend to be more mutable as they refer to practice arrangements, referral procedures, service coordination, and other organizational actions. Using electronic medical records (EMRs) as an example, the number of computer workstations and types of software may be described as elements of organizational structure, but the ways in which they are used to deliver care (e.g., decision support capacities, communication processes between providers) represent organizational processes underlying health information technology [56].

The role of *culture and relationships as organizational attributes *also are important to health care redesign and implementation of evidence-based practice [57]. Schein has defined culture as a pattern of shared basic assumptions that groups learn as a function of the problems they solve in response to external adaptation and internal integration [58]. When these group assumptions have worked well enough to be considered valid, they are taught to new members as the correct way to think and feel in relation to those problems (i.e., "This is how things are done around here") [58,59]. As is often the case, evidence-based practice is likely to reflect a new way of doing things, and thus may come into conflict with the prevailing culture of a practice.

There are, however, highly divergent views on how to study culture [59,60]. Culture encompasses a wide range of concepts that capture attitudes, beliefs and feelings about how the organization functions or the role of the individual (or team) within the organization (e.g., leadership, practice autonomy, quality improvement orientation, readiness to change) [61,62]. Culture has been classified as both a structural feature or measurable organizational average that characterizes context or an explicit trait to accommodate, and an organizational process or symbolic approach for viewing the organizational life of an institution [57,63]. Integral to the evaluation of and adaptation to local culture is the need to understand and appreciate the dynamics of relationships within and outside health care organizations that influence the adoption and use of innovations [64,65]. These dynamics may include consequences of political and social ideologies that may exert themselves on what is acceptable organizational behaviour [63]. Organizational culture is hypothesized to influence operational effectiveness, readiness to adopt new practices, and professional behaviour and style, and is considered by many to be a critical determinant of organizational performance [33,37]. Thus, culture change is commonly treated as an explicit (or implicit) part of efforts to implement evidence-based practice, insofar as QI interventions aim to change business as usual [66-68]. Despite substantial interest in the potential of culture as an organizational attribute, there is no widely agreed upon instrument to measure culture – and no consensus on how best to analyze or apply findings from these data to improve implementation of evidence-based practice. Also, organizational culture as measured among VA employees has been fairly consistent over time, raising issues about its mutability and the measures' sensitivity to change.

*Organizational outcomes *are akin to other measures of quality at the provider or patient level, with the exception that they are best expressed as the aggregation or roll-up of processes or outcomes at the organizational level. While the unit of analysis may differ (e.g., team, clinic, practice, hospital, system), organizational outcomes are often reflected as performance measures or practice patterns that serve as summary measures of process quality (i.e., the percentage of eligible diabetics receiving foot sensation exams) or intermediate outcomes (i.e., glycemic control among all diabetics in the entire practice). Other outcomes include disease-related outcomes (e.g., complication rates, disease-specific morbidity and mortality), practice-level or population-based measures of effectiveness (e.g., ambulatory care sensitive admission rates, functional status), utilization patterns and costs. Many trials and observational studies of the implementation of evidence-based practice continue to focus on "enrolled" populations rather than the entire practice that would be likely to experience the new care model or practice intervention under routine conditions. Organizational outcomes are distinct only insofar as they represent what the entire practice or institution would experience as a whole once implementation is complete, and are thus inter-related to other evaluation activities.

## The role of organizational research in the QUERI model

One of the foundations of QUERI has been to help operationalize the "interdependent relationships among clinicians, managers, policy makers, and researchers" [69].

The VA QUERI program's progress in conducting a series of progressively larger, multi-site implementation studies brings the nature and importance of organizational factors and the need for related planning into rapid relief. While most efforts outside the VA have focused on only a few and often immutable organizational parameters, such as size, QUERI studies have been able to uniquely capitalize on the size and diversity of the VA health care system to integrate organizational research more systematically. Therefore, the role of organizational research is both to understand the changeability of organizational attributes and, when fixed, to integrate them as modifiers in analyses of the effectiveness and impact of implementation efforts.

In the following sections, we describe the organizational research considerations that parallel the QUERI steps (Table 3) and describe examples of QUERI applications for each step (Table 4).

**Table 3:** The role of organizational research in QUERI

**6-step QUERI process**	**Role of organizational research**
	
	**Organizational (or practice) level**
**#1: Select diseases/conditions/populations:** • Identify and prioritize high-risk/high-burden clinical conditions • Identify high-priority clinical practices/outcomes within a selected condition	• Evaluate disease prevalence among member organizations or individual practices to ascertain how salient target conditions are system-wide (i.e., related to organizational readiness to change)

**#2: Identify evidence-based guidelines and clinical recommendations:** • Identify evidence-based practice guidelines • Identify evidence-based clinical recommendations	• Begin to consider implications of organizational settings where efficacy and effectiveness studies were conducted vs. where evidence will subsequently be applied

**#3: Measure and diagnose quality gaps:** • Measure existing practice patterns and outcomes across VHA, identify variations • Identify determinants of current practices • Diagnose quality gaps and identify barriers and facilitators to improvement	• Measure general organizational determinants of variations relative to the targeted condition/practice • Include measures of organizational structure and processes when diagnosing quality gaps • Determine general organizational factors that serve as barriers and facilitators to improvement to implementation in general and specific to the targeted condition/practice

**#4: Implement improvement programs **(strategy, program, program components or tools) to address quality gaps • Identify QI interventions (e.g., per literature reviews) • Develop or adapt QI interventions (e.g., educational resources, decision support) • Implement QI interventions	• Assess/diagnose local needs, gaps, and capacities in target sites • Use organizational characteristics to facilitate site selection for implementation • Evaluate organizational readiness to change • Design and evaluate additional intervention components based on local context (tailoring)

**#5: Evaluate improvement programs** • Assess improvement program feasibility, implementation, and impacts on patient, family and system outcomes	• Determine organizational facilitators that may be leveraged (e.g., leadership support) and barriers that may be amenable to resolution during the study (e.g., non-supportive process) or that may aide interpretation of findings
	
**#6: Evaluate improvement programs** • Assess improvement program impacts on health-related quality of life (HRQOL)	• Evaluate organizational structure, process and behaviours related to adoption and penetration • Analyze site and system-level effects and costs • Inform policy development for sustainability and spread to different organizational types and levels of complexity

**Table 4:** Examples of QUERI organizational research findings and their application in QUERI implementation research

**QUERI Center**	**Condition**	**Examples of steps #1–3 organizational research**	**Selected QUERI applications to steps #4–6**
Mental Health (MH) QUERI	Depression	• Guidelines adapted for local use taking organizational resources and priorities into account • Assessed national sample of PC clinics to understand variations in structure and processes of care (e.g., PC-based vs. referral focus) • Used national organizational survey to measure factors associated with PC-MH joint management of depression: • practice size (small-to-medium size) • more generalist MDs (vs. MD extenders) • greater specialty access (vs. pre-authorization for specialty use) • higher PC practice autonomy and provider incentives • Guidelines updated based on lessons learned from new randomized trials (Steps #4–6 full circle to revise Step #1)	• Used knowledge of organizational factors (Step #3) to select 1^st ^generation sites for implementing collaborative care (e.g., small-to-medium size sites with evidence of joint PC-MH management) • Measured site-specific structure using interviews of PC and MH leaders • Used site variations to target additional intervention resources to sites needing more provider education to ensure formulary access to antidepressants • Adapted intervention to accommodate staffing constraints (e.g., use of telephone vs. on-site care manager) • Identified organizational factors associated with adoption/penetration of collaborative care (e.g., sites with greater autonomy tend to push intervention to more providers faster but have greater difficulty sustaining it than sites that take more time to adapt the intervention among smaller provider groups). • Applied organizational factors to further adapt implementation for rollout to 2^nd ^generation sites

Substance Use Disorders QUERI	Smoking cessation	• Used national organizational survey to measure factors associated with higher tobacco counselling rates: • small-to-medium non-academic VAs • sites with greater staff commitment to QI • sites with integrated nurse practitioners and behavioural health professionals in PC practice • sites with separate PC budgets • sites with inpatient-outpatient continuity	• Used site surveys and administrative data to ascertain organizational resources before introducing evidence-based options (e.g., PC-based changes in care vs. specialty referral-based changes) • Used organizational factors to pair PC practices on size and academic affiliation in group randomized trial • Measured site-specific structure during and after implementation using key informant organizational surveys • Adapted intervention to accommodate local structural variations (e.g., added pharmacotherapy training) • Redesigned intervention to address factors that hindered adoption (e.g., telephone counselling)

	Alcohol use disorders	• Used national organizational survey to evaluate factors associated with PC management of alcohol use: • sufficiency of PC clinical support arrangements • physician involvement in QI • statistician for decision support • PCP responsibility for chronic care • availability of seminars on cost-effective care	• Combined organizational surveys of VA primary care practices and substance use programs to evaluate availability of alcohol treatment programs • Further organizational research planned before design and implementation of QI interventions

Colorectal Cancer QUERI	Colorectal cancer (CRC) screening	• Measured system capacity for colonoscopy using key informant organizational survey: • availability of/access to GI specialists • key coordination mechanisms between PC-GI needed • Used national organizational survey to evaluate factors associated with higher CRC screening rates: • PC practice autonomy • sufficiency of clinical practice support arrangements in PC practice • smaller PC practices	• Implementation of new organizational supports for obtaining colonoscopies for patients with +FOBT • Evaluated interaction between organizational and patient-level factors (e.g., racial-ethnic/gender differences) • Measured CRC-specific organizational factors (e.g., GI staffing, use of PC-GI service agreements, use of community providers) to inform intervention design • Integrated GI staffing and other organizational variables into system-level VA cost-effectiveness model

HIV/Hepatitis QUERI	HIV disease	• Categorized VA facilities based on: • HIV caseload • Use of HIV guidelines • Methods of promoting adherence (e.g., chart audits, feedback) • Used national HIV organizational survey to measure HIV care variations: • Most urban VAs have special HIV clinics staffed with experienced HIV providers; rural VAs tend to manage HIV in PC, use outside experts • Most VAs have 1+ HIV case manager • Used national organizational survey to measure organizational readiness for change, local barriers and preferences for different types of QI implementation	• Used organizational care arrangements from national survey to select sites for trial (i.e., minimum eligibility criteria) (e.g., adopted HIV QI guidelines, reported provider readiness for change) • Evaluated organizational factors associated with adoption of HIV guidelines (e.g., urban, complex, larger HIV caseloads, use HIV case managers, fewer barriers to antiretroviral therapy and opportunistic infection prophylaxis guidelines) and HIV-related QI (e.g., larger, more complex facilities) • Used administrative data to classify VA facilities by level of organizational attributes of HIV care and analyzed links to better control of HIV infection

Diabetes QUERI	Diabetes mellitus	• Used organizational surveys to benchmark VA practices with those outside the system • Appraised performance variations at the patient, provider and facility levels • Used organizational surveys to identify factors associated with glycemic control: • Greater PC authority over establishing clinical policies • Greater staffing authority • Greater use of computerized diabetes reminders • Special teams or protocols to respond to clinical issues • Weekly multidisciplinary clinical team meetings	• Used PC provider survey to study influences of organization of care and provider training on treatment of pain among diabetics (e.g., inadequate training in chronic pain management, treatment of pain conditions perceived as beyond provider's scope of experience) • Evaluating clinician, organizational and patient factors contributing to failure to change therapy when blood pressure among diabetics is elevated

### Evaluate disease burden and set organizational priorities (Step #1)

In a national health care system like the VA, conditions have been chosen on the basis of nationally prevalent conditions (e.g., diabetics, depression) or those associated with high treatment costs (e.g., HIV/AIDS, schizophrenia). Target conditions also have been updated periodically to accommodate changes over time (e.g., additional focus on hepatitis C added to the QUERI-HIV/Hepatitis Center's mission and scope).

On a national level, all VA facilities have commonly been held to the same performance standards regardless of organizational variations in caseload or resources. In smaller systems or independent health care facilities, organizational priorities should be established based on ascertainment of disease burden at the appropriate target level (e.g., individual practices or clusters of practices). At this step, it is important to determine how salient target conditions are among member organizations or individual practices by evaluating the range or variation in disease burden or performance. Modified Delphi expert panel techniques have been useful in establishing consensus among various organizational stakeholders in order to set institutional priorities [70]. These techniques entail advance presentation of the evidence base for a particular condition or setting (e.g., compendium of effective interventions based on systematic reviews) [71,72], as well as stakeholders' pre-ratings of their perceptions of organizational needs and resources, followed by an in-person meeting where summary pre-ratings are reviewed and discussed. Participants then re-rate and prioritize planned actions with the help of a trained moderator.

Many QUERI efforts have benefited from inclusion of QUERI-relevant measures in the national VA performance measurement system (e.g., glycemic control, colorectal cancer screening). This alignment of QUERI and national VA patient care goals fosters research/clinical partnerships in support of implementing evidence-based practice. For those QUERI centers whose conditions fall outside the national performance measurement system (e.g., HIV/AIDS), alternate strategies, such as business case modelling (i.e., spreadsheet-type models summarizing operational impacts of deploying a new care model or type of practice), have anecdotally met with some success.

### Identify evidence-based practice guidelines and clinical recommendations (Step #2)

Organizational attributes have come into play at Step #2 in QUERI, when established guidelines assume access to or availability of certain organizational resources to accomplish them (e.g., specialty access, equipment availability). Many guidelines do not contain recommendations that consider organizational factors. It is thus essential to begin to consider the implications of the differences between the characteristics of the health care organizations in which efficacy and effectiveness have been established vs. those in which the evidence-based practices will subsequently be applied in order to improve their reach and adoption [73].

For example, for the Colorectal Cancer QUERI, VA and the U.S. Department of Defense (DoD) guidelines for colorectal cancer screening were updated with recommendations for direct colonoscopy as the screening test of choice. Implementation of evidence-based practice in these circumstances would require different approaches in VA facilities with adequate in-house gastroenterology staffing compared to those where specialty access required referral to another VA facility or to community resources to accomplish the same goal. Anecdotally, in the face of limited specialty resources, some VA facilities adapted guideline adherence policies by fostering primary care-based sigmoidoscopies. In contrast, the U.S. Public Health Service smoking cessation guidelines relied on by researchers in the Substance Use Disorders QUERI offer a more explicit roadmap that includes adaptive changes to health care settings to promote adherence, with options for actions within and outside of primary care [74]. However, even they are limited in terms of their guidance on how best to accommodate different organizational constraints.

### Measure and diagnose quality/performance gaps (Step #3)

The inclusion of organizational research in Step #3 has had particular value. For example, Colorectal Cancer QUERI researchers have evaluated the organizational determinants of variations in colorectal cancer screening performance as an early step prior to designing implementation strategies [75]. They also assessed system capacity to determine how implementation strategies might need to be adapted to deal with specialty shortages or referral arrangements [13]. Therefore, organizational knowledge from Step #3 studies may be used to facilitate planning for Step #4 implementation efforts.

Several QUERI centers have capitalized on existing organizational databases, while others have collected their own QUERI-specific organizational structure and process data for these purposes. These efforts have enabled QUERI researchers to document variations in how care is organized across the system, benchmark it with other systems, elucidate organizational factors associated with adoption of guidelines and quality improvement activities, and explicitly integrate these local variations into the design and conduct of implementation approaches (Table 4) [76-82].

### Implement quality improvement (QI) interventions (Step #4)

Organizational factors come into play throughout the process of developing, adapting and implementing QI strategies for implementing research findings into routine care (Table 4). They provide a framework for diagnosing critical local conditions; developing a general implementation strategy; creating specific accommodations for different organizational contexts; and informing the design of subsequent evaluation studies. For example, in preparing to implement evidence-based interventions, it is important to assess local needs and capacities. Such needs assessments include appraisals of organizational readiness to change and diagnosis of system barriers and facilitators to the adoption of evidence-based practice at target sites [13].

The degree to which QUERI researchers have used information about organizational variations in the design and implementation of QI interventions has varied (Table 4). Organizational factors sometimes informed site selection for participation in large-scale implementation studies (e.g., Mental Health QUERI) [77,83,84]. They also were used as a foundation for the accommodation of local organizational characteristics through adaptation of intervention components (i.e., addition, elimination or modification).

Few large-scale experimental trials of the effects of specific adaptations to local organizational context that may be incorporated in Step #4 implementation efforts have been conducted. Recruitment of a sufficient number of organizations with the characteristics of interest typically requires dozens of health care settings, adding to the size, expense and complexity of cluster randomized trials [85]. Therefore, adaptation or tailoring of an implementation strategy's components to local organizational context commonly occurs as extrapolations from associations identified in quantitative cross-sectional analyses – or through application of qualitative data (Table 4). It is important that the level of evidence supporting on-the-ground changes in implementation protocols and procedures from site-to-site be clearly described. Otherwise, our ability to evaluate their deployment of these adaptations is limited.

### Evaluate quality improvement (QI) interventions (Steps #5–6)

Consideration of organizational factors should explicitly shape the evaluation methods used in Steps #5 and #6 (Table 4). Methods used for assessing organizational factors in these types of evaluations use multi-method techniques, commonly combining qualitative inquiry (e.g., semi-structured interviews of key informants or focus groups of providers) and quantitative data collection (e.g., through surveys of leaders, providers or patients).

Unlike the organizational variations studies described for Step #3 or the adaptation or addition of program components that address organizational context in Step #4, QUERI studies in Steps #5 and #6 explore the organizational factors associated with adoption, implementation and impacts of the targeted QI intervention (Table 4). These studies may be distinguished from the pre-implementation organizational research (which is chiefly cross-sectional) in that implementation researchers aim to evaluate organizational predictors of quality improvement (i.e., *changes in quality *post-implementation). This is related to the more action-oriented research where fewer organizational factors are controlled for and also to pragmatic randomized trials where sufficiently large samples of organizations are included to enable subgroup analyses, as with different practices. Here, organizational evaluation may be formative (i.e., iterative component of practice redesign efforts) and outcomes-oriented (e.g., cluster randomized trials of implementation strategies or new policies or procedures designed to improve care); within QUERI, these evaluation approaches co-occur [45,85,86]. They also may focus on the organizational factors associated with adoption, penetration, sustainability or spread of interventions that have already been shown to be efficacious under ideal circumstances and effective in different types of settings.

Organizational research at Steps #5–6 has focused either on explicit integration and evaluation of organizational factors within the QI strategy itself (e.g., adding organizational supports as recommended in the U.S. Institute of Medicine [IoM] report) [87], or evaluation of organizational influences on how well a QI strategy performed across intervention sites (Table 4). Understanding site-level effects and provider variation similarly enable refinement and improved fit of the evidence to local organizational and practice issues [88-90].

Several QUERI examples apply. For example, in the Substance Use Disorders (SUD) QUERI, a process evaluation of organizational barriers in a multi-state group randomized trial of evidence-based quality improvement strategies for implementing smoking cessation guidelines led to a redesign of key intervention components (Table 4). During the trial, qualitative evaluation of organizational processes identified patient reluctance to attend smoking cessation clinics, inconsistent provider readiness to counsel in primary care, and variable ease in referral and capacity in behavioural health sessions [91]. Quantitative surveys and analysis of the organizational factors (e.g., formulary changes, smoking cessation clinic availability) influencing smoking cessation clinic referral practices across the 18 participating sites also were conducted [92,93]. The new implementation strategy – deployed in a subsequent trial – replaced the need for multiple in-person counselling sessions with EMR-based referral to telephone counselling. The Mental Health QUERI has used similar methods to implement depression collaborative care in increasingly diverse practices. With a parallel focus on schizophrenia, the Mental Health QUERI also has done extensive work using EMR automated data to monitor antipsychotic prescribing as a tool for QI evaluation in different locales [94]. Each QUERI center is working through these types of organizational research issues as implementation efforts accelerate throughout the VA.

## Discussion

We posit that a better understanding of the organizational factors related to implementation of evidence-based practice is a critical adjunct to efforts to systematically improve quality across a system of care, especially when the evidence must be translated to increasingly diverse practice settings. Specifically, more explicit accommodation of organizational inquiry into implementation research agendas has helped QUERI researchers to better frame and extend their work as they move toward regional and national spread activities. While some QUERI researchers have used traditional or pragmatic randomized trials, they also have worked to integrate complementary evaluation methods that capture organizational attributes in ways that enable them to open the "black box" of implementation, and in turn help inform and accelerate adoption and spread of evidence-based practice in each successive wave of practices. We argue for the value of casting organizational research as one of several lenses through which implementation research may be viewed.

Systematically integrating organizational research applications into implementation research is not without its challenges. Organizational research comes with its own methodological challenges in terms of appropriate study designs, adequate statistical power at the organizational unit of analysis, and multi-level analytical issues that require attention. Integrating organizational factors into empirical research has been daunting for most researchers given the logistical difficulties and costs of working with large numbers of hospitals or practices [95]. However, even in smaller studies, it is not uncommon for researchers to describe the effectiveness of interventions, such as reminders or audit-and-feedback, without describing the organizational supports or other contextual factors influencing their success [3]. No less important, the ability to study and manipulate organizational factors is confounded by sample size requirements of traditional research designs, invoking serious limitations in the conduct of most organizational research. Measurement of organizational constructs also can be difficult and requires identifying appropriate data sources (e.g., administrative data, practice checklists, surveys) and the right respondent(s) at one or more levels of the organization as key informants, if primary data are to be collected. Just as research at the patient or provider level tends to disregard organizational factors, organizational research also should adequately account for the contribution of patient characteristics (e.g., socio-demography, health status, clinical severity, co-morbidity) and provider characteristics (e.g., knowledge, attitudes, behaviour), where possible. Unfortunately, patient-level data clustered within providers and their respective organizations are not commonly available, creating built-in limitations in the interpretability of organizational research.

While this paper focuses on the influence of internal organizational characteristics on implementation of evidence-based practice, recognition of the importance of context requires brief mention of environmental factors (i.e., characteristics external to the organization). Environmental factors, defined as characteristics external to the organization, include geography (e.g., region, state, urban/rural location), area population characteristics (e.g., population density, socio-demography, community health status), area resources (e.g., numbers of health care providers per 1,000 residents), and other relevant area characteristics (e.g., managed care penetration, regulatory environment). Such factors may influence how health care organizations are structured, though organizational factors also may serve to mediate the impact of environmental factors on care processes and patient outcomes. For example, higher primary physician-to-patient staffing ratios in rural VA facilities appear to offset local gaps in specialty access and are associated with comparable quality [96]. Not surprisingly, deployment of system interventions into urban vs. rural facilities, often dictates different organizational adaptations to account for area resources. Explicit acknowledgment and planning for these influences ahead of implementation efforts is arguably a better approach than post-hoc reactions once in the field. The key is that context matters and requires continual evaluation to determine how context may constrain or create opportunities for improving implementation [97].

The VA's investment in QUERI has helped advance knowledge about the role of organizational factors in implementation. For example, organizational size appears to operate differently for different types of QI interventions. While organizational size is a positive factor for less complex QI interventions (i.e., where slack resources may be brought to bear), medium-sized facilities appear to be more nimble when facing the challenges of implementing more complex organizational changes (e.g., introduction of a new care model). In contrast, if practices were too small, they suffered from inadequate staffing and limited local autonomy for decision-making (i.e., had to wait for direction, were not able to identify a local champion). If they were too large, they suffered organizational inertia or required more organizational supports for coordination across departments or services. These barriers were sometimes overcome with sufficient leadership support and allocation of additional resources. Organizational control of those resources also is important. In the VA, like other large health care systems, resource control was sometimes one or more levels above the practice in which the QI intervention was being implemented. This required negotiation with senior leaders with varying levels of awareness and understanding of frontline needs or culture, and repeated marketing messages to different stakeholders at each level. Control of how care was organized also was important but did not always operate in expected ways. Practice autonomy emerged as a facilitator of more rapid implementation (i.e., faster penetration among providers in a practice); however, their speed appeared to undermine sustainability. Further work is needed to validate these findings for more QUERI conditions among increasingly diverse practice settings and in organizations outside the VA. For example, do the same findings hold true for depression as they do for diabetes? Varying levels of supporting evidence were noted for many organizational structures and processes in relation to quality of implementation. While the VA is most generalizable to large health systems, including U.S. regional systems like Kaiser Permanente and national health systems, such as those in the UK and Australia [98], many of the organizational factors studied also have correlates in smaller practices.

At this juncture, QUERI implementation research studies are progressing from local to regional to national in scope [12]. In parallel, methodologically – and along the lines of the QUERI steps – they are moving from variations studies to tests of intervention and implementation effectiveness to evaluations of spread, and then to policy development [13]. It is incumbent on us to contribute to bridging the gap between research and practice by considering the potential for accelerating implementation success by explicitly addressing organizational factors in our work.

## List of abbreviations used

VHA: Veterans Health Administration; QUERI: Quality Enhancement Research Initiative; EMR: electronic medical record; CPRS: Computerized Patient Records System; CQI: continuous quality improvement; QI: quality improvement; PC: primary care; GI: gastrointestinal; HIV: human immunodeficiency virus.

## Competing interests

The author declares that she has no competing interests.

## Authors' contributions

EMY conceived of the content, identified relevant work, and drafted, iteratively revised, and finalized the manuscript and then reviewed and approved the final version.
